# An EEG-/EOG-Based Hybrid Brain-Computer Interface: Application on Controlling an Integrated Wheelchair Robotic Arm System

**DOI:** 10.3389/fnins.2019.01243

**Published:** 2019-11-22

**Authors:** Qiyun Huang, Zhijun Zhang, Tianyou Yu, Shenghong He, Yuanqing Li

**Affiliations:** ^1^Center for Brain Computer Interfaces and Brain Information Processing, South China University of Technology, Guangzhou, China; ^2^MRC Brain Network Dynamics Unit, Nuffield Department of Clinical Neurosciences, University of Oxford, Oxford, United Kingdom

**Keywords:** brain-computer interface (BCI), hybrid BCI, electroencephalogram (EEG), electrooculogram (EOG), wheelchair, robotic arm

## Abstract

Most existing brain-computer Interfaces (BCIs) are designed to control a single assistive device, such as a wheelchair, a robotic arm or a prosthetic limb. However, many daily tasks require combined functions which can only be realized by integrating multiple robotic devices. Such integration raises the requirement of the control accuracy and is more challenging to achieve a reliable control compared with the single device case. In this study, we propose a novel hybrid BCI with high accuracy based on electroencephalogram (EEG) and electrooculogram (EOG) to control an integrated wheelchair robotic arm system. The user turns the wheelchair left/right by performing left/right hand motor imagery (MI), and generates other commands for the wheelchair and the robotic arm by performing eye blinks and eyebrow raising movements. Twenty-two subjects participated in a MI training session and five of them completed a mobile self-drinking experiment, which was designed purposely with high accuracy requirements. The results demonstrated that the proposed hBCI could provide satisfied control accuracy for a system that consists of multiple robotic devices, and showed the potential of BCI-controlled systems to be applied in complex daily tasks.

## 1. Introduction

An Electroencephalogram (EEG)-based brain-computer interface (BCI) records electrical signals of brain cells from scalp and translates them into various communication or control commands (Wolpaw et al., 2000). Common modalities used in EEG-based BCIs include steady-state visual evoked potentials (SSVEP) (Cheng et al., 2015), event-related potentials (ERPs) (Blankertz et al., 2011; Jin et al., 2017), and mu (8–12 Hz)/beta (18–26 Hz) rhythms related to motor imagery (MI) (Lafleur et al., 2013).

A main focus of the EEG-based BCIs is to combine them with existing assistive devices, such as a prosthesis or a wheelchair, to support motor substitution of the user's limb functions, e.g., the grasping function and the walking function (Millán et al., 2010). While SSVEP- and ERP-based BCIs only provide discrete commands, MI-based ones can generate nearly continuous outputs in real time, which makes them a good fit for manipulating assistive devices that require highly accurate and continuous control. Several purely MI-based BCIs have been developed to realize basic control of external devices (Wolpaw and McFarland, 2004; Lafleur et al., 2013; Meng et al., 2016). However, MI-based BCIs still suffer from limited number of distinguishable MI tasks (Yu et al., 2015).

To overcome the limitation of using a single MI paradigm, many excellent works have been established in recent years to realize multidimensional control of external devices by combining the MI with other EEG modalities (Rebsamen et al., 2010; Long et al., 2012; Li et al., 2013; Bhattacharyya et al., 2014; Ma et al., 2017) or other bioelectrical signals (Punsawad et al., 2010; Jun et al., 2014; Witkowski et al., 2014; Ma et al., 2015; Soekadar et al., 2015; Minati et al., 2016), i.e., using a hybrid brain-computer interfaces (hBCIs) (Pfurtscheller et al., 2010; Hong and Khan, 2017). For example, in Long et al. (2012) and Li et al. (2013) the user continuously controlled the direction (left/right turn) of a wheelchair using the left- or right- imagery, and used the P300 potential and SSVEP to generate discrete commands, such as acceleration/deceleration and stopping; in Ma et al. (2017), the users generated MI to control the moving of a robotic arm, and stop it by detecting the P300 potential.

Other than with different EEG modalities, MI can also be combined with other bioelectrical signals, such as Electrooculogram (EOG) signals and functional near infrared spectroscopy (fNIRS) (Khan and Hong, 2017), to build a hBCI. EOG signals are generated by eye movements and usually maintain a higher signal-to-noise ratio (SNR) compared with EEG signals (Maddirala and Shaik, 2016). In Witkowski et al. (2014), MI-related brain activities were translated into continuous hand exoskeleton-driven grasping motions which could be interrupted by EOG signals, aiming to enhancing the reliability and safety of the overall control. In Soekadar et al. (2015), Soekadar et al. demonstrated that the inclusion of EOG in a MI-based hand exoskeleton system could significantly improve the overall performance across all participants.

Prior studies have well-demonstrated the feasibility of using an hBCI to control a single assistive device, such as a wheelchair or a robotic arm. However, it is still unknown whether multiple devices can be integrated together and controlled by a single hBCI. Such integration is challenging because it requires higher control accuracies (i.e., the positional accuracy and the angular accuracy) and more control degrees than a single BCI-controlled device system. Also, the time and efforts consumed to control an integrated system are usually higher than that of any of its single component, which may reduce the reliability. In this study, we integrate a wheelchair and a robotic arm into a unified system, aiming to help the user move from a random place to approach and grasp a target object which is also randomly placed far away from the user. A novel hBCI based on EEG (the MI paradigm) and EOG signals is proposed to control the system. Specifically, for the wheelchair, users can continuously steer the wheelchair left/right by imagining left/right hand movements. Users generate discrete wheelchair commands, such as moving forward and backward and stopping, by implementing eye blinks and eyebrow movements. For the robotic arm, the eye blinks and eyebrow movements are utilized along with two cameras in a shared control mode. There were 22 healthy subjects participated in a MI training session, after which five of them (with accuracy over 80%) were asked to complete a tricky self-drinking task using the proposed system. The experimental results showed that the proposed hBCI could provide satisfied accuracy to control the integrated system and had the potential to help users complete daily tasks.

The remainder of this paper is organized as follows: section 2 is the methodologies, including the signal acquisition, the system framework and the hBCI; sections 3 and 4 describe the experiments and present the results; further discussions are included in section 5; and section 6 concludes the paper.

## 2. Methods

### 2.1. Signal Acquisition

**Figure 1:**
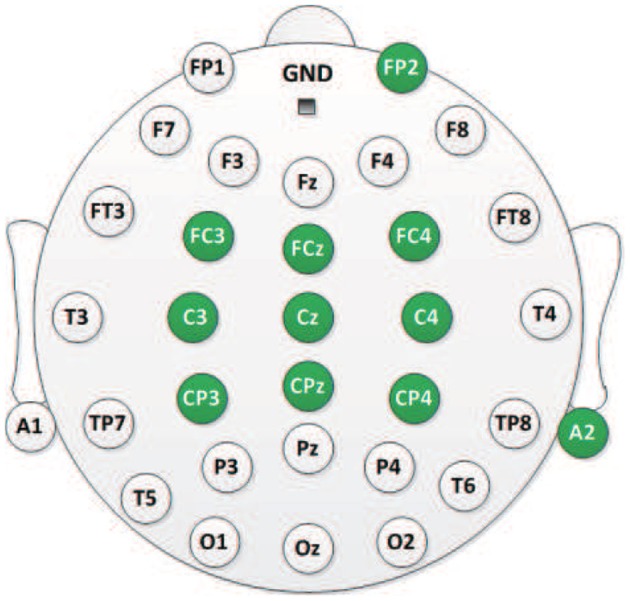
The 10–20 electrode distribution of a 32-channel Quik-cap. Eleven electrodes (green color) are employed in this study.

As shown in Figure 1, the EEG signals are recorded from nine electrodes (“FC3,” “FCz,” “FC4,” “C3,” “Cz,” “C4,” “CP3,” “CPz,” and “CP4”) attached on a 32-channel Quik-cap and amplified by a SynAmps2 amplifier [Neuroscan Compumeidcs, USA] with a sampling rate of 250 Hz. One electrode attached on the forehead (“FP2”) is used to record the EOG signals which are resulted from eye movements. The amplifier is grounded on the forehead, and “A2” is the reference electrode which is placed near the right ear lobe. The impedances between the scalp and all electrodes are maintained below 5 kΩ.

### 2.2. System Components

**Figure 2:**
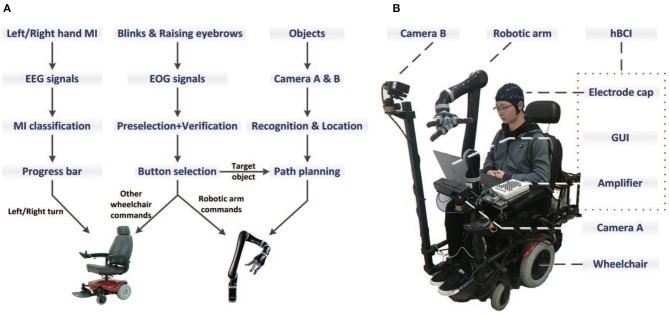
The system flowchart **(A)** and the basic components **(B)** which include the hBCI, a wheelchair, a six-degree intelligent robotic arm and two motion-sensing cameras.

The system consists of into two parts: (i) the control unit; and (ii) the execution unit. The control unit is a novel hBCI that processes the recorded EEG and EOG signals and translates them into various control commands. The execution unit is an integrated wheelchair robotic arm system which was built to help paralyzed people in Huang et al. (2019). As shown in Figure 2B, the hardware components include a laptop to present the GUI, a wheelchair [0.8 × 0.6 m, UL8W, Pihsiang Machinery Co. Ltd.], a six-degree intelligent robotic arm [JACO6 DOF-S, Kinova Robotics] and two motion-sensing cameras [Kinect v2, Microsoft].

### 2.3. GUI and Control Strategy

**Figure 3:**
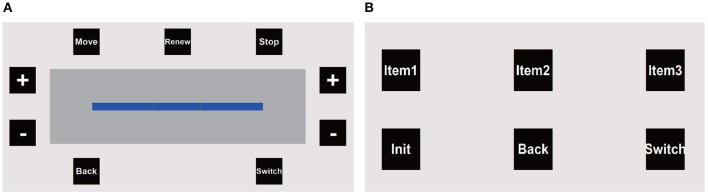
The GUI of the proposed hBCI consists of two separate panels: the wheelchair panel **(A)** and the robotic arm panel **(B)**.

The graphical user interface (GUI) of the hBCI consists of two separate panels: (i) the wheelchair panel (Figure 3A); and (ii) the robotic arm panel (Figure 3B). When the system is turned on, the wheelchair panel is presented. As shown in Figure 3A, the progress bar is used to control the wheelchair direction. The value of the bar represents the classification result of the user's left-right MI imagery. Two green lines are set at somewhere on the left and right sides of the bar as the left and right threshold, respectively. Initially, the value of the progress bar is 0 and the bar stops in the middle. The user can grow the bar to the left/right side by continuously imagining left/right hand movement. As long as the value of the bar exceeds the left/right threshold, the wheelchair is continuously turned to the left/right at an angular velocity of 0.1π/*s* (18°/s) (see details in the *EEG signals processing* section).

In the wheelchair panel, there are nine buttons placed around the progress bar that flash one by one in a predefined sequence. The interval between the onset of two continuous button flashes is 100 ms. Thus, the period of a complete round (i.e., each button flashes once) is 900 ms. To select a target button, the user first performs an intended blink in response to a flash of the target button. The system detects the intended blink and pre-selects a potential target button according to the timing of blinking. Next, if the pre-selected button is correct, the user needs to raise his/her eyebrows once to verify it. Only when a button is pre-selected and verified can the corresponding command be triggered (see details in the *EOG signals processing* section). The “Move” and “Back” buttons represent moving forward and backward at 0.2 m/s, respectively. The “Stop” button is used to stop the moving and turning of the wheelchair immediately. Other buttons in the wheelchair panel are active only when the wheelchair is stopped. For example, the user can increase/decrease the left and right threshold values by selecting the “+”/“−” buttons on the left and right sides, and renew the MI classification parameters (see details in the *EEG signals processing* section). The “Switch” button is used to switch the GUI to the robotic arm panel.

In the robotic arm panel, there are 6 buttons which flash one by one with an interval of 150 ms, as shown in Figure 3B. Thus, the round period is also 900 ms. The three object buttons (“Item 1,” “Item 2,” and “Item 3”) represent three target objects that can be grasped. Once the user selects an object button, the two cameras (Camera A and B) would return the coordinates of the object as well as the user's mouth to the robotic arm, and then the arm automatically plan the path to grasp the target and bring it to the user's mouth. The “Init” button is used to initiate the arm's internal parameters and move it to the home position. After the target has been brought to the mouth, the user can select the “Back” button to ask the arm to put the target back automatically. The “Switch” button is used to switch to the wheelchair panel. The system flowchart is illustrated in Figure 2A.

### 2.4. EEG Signals Processing

A supervised machine learning process was implemented to process the multichannel EEG signals, which included two parts: (i) the offline model training process; and (ii) the online classification process. In the offline model training process, each user was asked to complete several left/right hand MI tasks. The recorded and labeled (left or right) EEG signals from the nine electrodes (“FC3,” “FCz,” “FC4,” “C3,” “Cz,” “C4,” “CP3,” “CPz,” and “CP4”) were first referenced with the signals from “A2.” Then, the signals were band-pass filtered around 8–30 Hz (α and β bands). For the feature extraction, the common spatial pattern (CSP) method was applied. Specifically, a covariance matrix was achieved by the following formula:

(1) $$\begin{array}{l} R_{i}=\frac{X_{i}\times {X_{i}}^{T}}{trace\left(X_{i}\times {X_{i}}^{T}\right)} \end{array}$$

where $X_{i}\in R^{M\times N}$ denotes the filtered EEG data matrix of the *i*_*th*_ trial, *M* is the number of channels (9 in this case), *N* is the number of samples in each trial.

Then, the covariance matrixes that belong to the same class (left or right) were added up as *SUM*_*l*_ or *SUM*_*r*_. The goal of CSP was to find a spatial filter *W* ∈ *R*^*m*×*N*^ (*m* is the order of the spatial filter) that maximized the band power difference between *SUM*_*l*_ and *SUM*_*r*_, and this *W* could be constructed using the eigenvectors of *SUM*_*l*_ and *SUM*_*r*_ (Li and Guan, 2008). The MI feature used in this study was defined in MATLAB as below:

(2) $$\begin{array}{l} F_{i}=log\frac{diag\left(W\times R_{i}\times W^{T}\right)}{sum\left(diag\left(W\times R_{i}\times W^{T}\right)\right)} \end{array}$$

where $F_{i}\in R^{m\times m}$ denotes the MI feature of the EEG data of the *i*_*th*_ trial. Further, the features of all trials and their labels were used to learn a MI classifier based on the support vector machine (SVM) algorithm.

In the online classification process, the MI classifier evaluated a 2-s real-time EEG data epoch every 0.2 s, and generated a score *c*, which represents the comparative similarity between the input data epoch and the two classes. The mean score for the idle state (i.e., when the user did not imagine) was termed as the idle score *C*_*idle*_. Each newly generated score *c* was compared to *C*_*idle*_, and the result was used to steer the wheelchair as below:

(3) $$\begin{array}{l} \text{Turn left:}\{\begin{array}{c} c<C_{idle}\\ \left|c-C_{idle}\right|>TH_{l} \end{array} \end{array}$$

(4) $$\begin{array}{l} \text{Turn right:}\{\begin{array}{c} c>C_{idle}\\ \left|c-C_{idle}\right|>TH_{r} \end{array} \end{array}$$

where *TH*_*l*_ and *TH*_*r*_ denote the left and right threshold, respectively. As mentioned in the *GUI and control strategy* section, once the “Renew” button was selected when the wheelchair was stopped, the system updated *C*_*idle*_ by averaging the scores of the next 3 s, during which the user was supposed to be in the idle state. If *C*_*idle*_ was renewed, the threshold values *TH*_*l*_ and *TH*_*r*_ also needed to be adjusted, which could be realized by selecting the “+”/“−” buttons on the wheelchair panel, as shown in Figure 3A.

### 2.5. EOG Signals Processing

**Figure 4:**
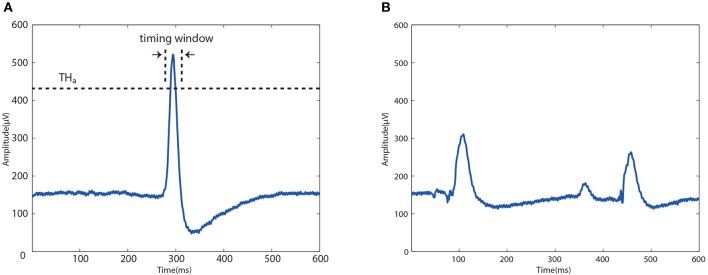
Typical EOG waveform of an intended blink **(A)** and unintended blinks **(B)**. The peak of the intended waveform should be located within a predefined timing window and pass an amplitude threshold *TH*_*a*_.

To select a button on the GUI, users were asked to perform two kinds of eye movements: one intended blink and one eyebrow raising movement. Specifically, after each button flash, the algorithm evaluated a 600-ms EOG data epoch (i.e., 150 samples) which started from the onset of that flash to examine whether it contains an intended blink. A blink was detected and recognized as intended if two conditions were satisfied: (i) The 600-ms data epoch passed a multi-threshold waveform check (as described in Huang et al., 2018), which implied that there was a blink waveform (either intended or unintended) contained in this epoch; (ii) The detected blink waveform was regarded as intended if it was occurred within a certain delay window after the flash onset, and also the peak of the waveform should passe an intended amplitude threshold, as shown in Figure 4. The second condition was based on experimental observations: although the response time to a flash varied among individuals, it was relatively stable for a particular user (e.g., 280–320 ms after the flash), and intended blinks usually had a higher amplitude than unintended ones due to the more strong eye movement. For example, if a blink waveform with enough amplitude was detected about 280 ms after the flash onset of “MOVE,” it would be recognized as an intended blink in response to “MOVE.” For other buttons, the delay should be extended or shortened by at least 100 ms due to the button flash interval.

However, there was still a possibility that an unintended blink was mistakenly detected to be intended. Thus, a verification process was implemented to further exclude the unintended inference. Specifically, when an intended blink was detected in the EOG epoch after a button flash, the system just preselected the button and highlighted it in blue as feedback without any command activated. The user was asked to judge the feedback and raise eyebrows to verify if it was what he/she wanted. Only when a button was preselected and verified, was the corresponding command triggered. The detection algorithm for the eyebrow raising movement was similar with the multi-threshold waveform check used in the blink detection, which aimed to recognize different eye movements by checking particular waveform parameters, such as the amplitude, the speed (i.e., the differential value) and the duration of the movement (Huang et al., 2019).

## 3. Experiments

Twenty-two healthy subjects (6 female and 16 males, aged between 22 and 37 years) participated in a MI-based training session and an EOG-based training session without actual control of the wheelchair and the robotic arm. The MI-based training session was designed to help subjects learn and improve the ability of voluntarily modulating the sensorimotor EEG in the motor cortex by performing left-right hand MI task; The EOG-based training session was supposed to help subjects learn how to select a flashing button using the proposed EOG paradigm. Next, five of the 22 subjects with satisfied performance were asked to complete a mobile self-drinking experiment. The experiments were approved by the Ethics Committee of Sichuan Provincial Rehabilitation Hospital. Written informed consent for experiments and the publication of individual information was obtained from all subjects.

### 3.1. MI-Based Training Session

All of the 22 subjects participated in a MI-training section on each of three different days in 2 weeks, each section consists of three sessions (i.e., nine sessions for each subject). Each MI-training session consisted of an offline run without feedback and an online run with feedback. In an offline run, the subjects performed 40 random left-/right-hand MI trials according to the cue presented on screen. A trial began with a 5-s rest period, in which subjects relaxed and remained in an idle state. Then, a fixation cross was presented at the center of the screen for 2 s, prompting subjects to concentrate on the upcoming task cue. After the cross disappeared, an arrow randomly pointed to either the left or the right was appeared for 5 s. Subjects were asked to imagine the movement of the left or right hand, as indicated by the cue arrow. The recorded EEG data were then used to build a classifier and calculate the offline MI classification accuracy based on a 10-fold cross validation process.

It has been reported that feedback paradigm can enhance MI training (Yu et al., 2015). Thus, after each offline run, subjects completed an online run with visual feedback. Specifically, the subject was asked to change the state of the progress bar by performing left-/right-hand MI task. The left/right MI thresholds were set properly in this session to separate the bar into left, middle (idle), and right parts, ensuring that the subject could effectively control the bar to switch between the three parts. Any out-of-control situation implied the need of adjustment in the rest MI-training sessions, such as adjustment of the imagined hand movement. Subjects with an offline MI classification accuracy over 80% and showed a good control effect of the progress bar were selected to participate in the following experiments.

### 3.2. EOG-Based Training Session

In this session, five of the 22 subjects with satisfied MI performance were selected to complete 3 EOG-based training sessions, each of which consisted of 30 trials. In a trial, a random target button on the wheelchair panel was first highlighted in blue for 2 s. Then, all buttons started flashing as described in the *GUI* section. Subjects were asked to perform blinks and eyebrow movements to select the target button as soon as possible. The break between two continuous trials was 2 s. After the three sessions, each subject was asked to keep in the idle state for 10 min during which he/she just relaxed. Indicators, such as the selection accuracy, the selection delay and the false positive rate (FPR), were calculated to evaluate the EOG performance of the system.

### 3.3. Mobile Self-Drinking Experiment

**Figure 5:**
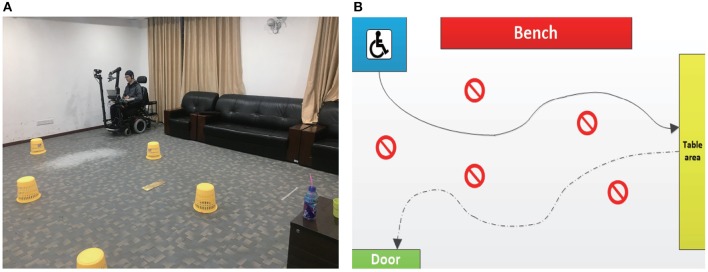
**(A)** The actual view of the experimental field. **(B)** A typical route that a subject (S1) drove through during the experiment.

Five of the 22 subjects that completed both the MI- and EOG-based training sessions participated in this experiment. As shown in Figures 5A,B, in an indoor experimental field (8 × 5 m), several obstacles were placed between the starting point and a randomly placed table, on which there were two different bottles with a straw and some water in each of them. To complete an experimental run, subjects were asked to control the system to complete three concatenated tasks: (i) Driving the system from the starting point to reach the table through the obstacles; (ii) Manipulating the robotic arm to grasp a target bottle, drink water with the straw and then put the bottle back; (iii) Driving the system to go through obstacles and a door (width: 1.15 m). Each subject completed three runs with the proposed hBCI.

## 4. Results

### 4.1. MI Training Results

In this study, each of the 22 subjects completed 9 MI training sessions. According to the binominal test theory, a significant statistical difference is supported if the *p*-value is smaller than 0.0056 (0.05/9). We use the following formula in MATLAB to calculate the *p*-value:

(5) $$\begin{array}{l} p\;=\;1-\;cdf{(}^{\prime }bino^{\prime },a,num,0.5) \end{array}$$

where *num* is the number of trials in a session (40 in this case), and *a* is the number of the correctly predicted trials in a session. By this formula, we can achieve that *a* should be larger than 27 to ensure that *p*-value is smaller than 0.0056. Thus, the smallest required number of correct trials in a session is 28, which means the accuracy is around 70% (28/40). Considering the high control precisions required in this study, we set 80% as a minimum passing accuracy to invite potential subjects to participate in more MI training sessions.

**Figure 6:**
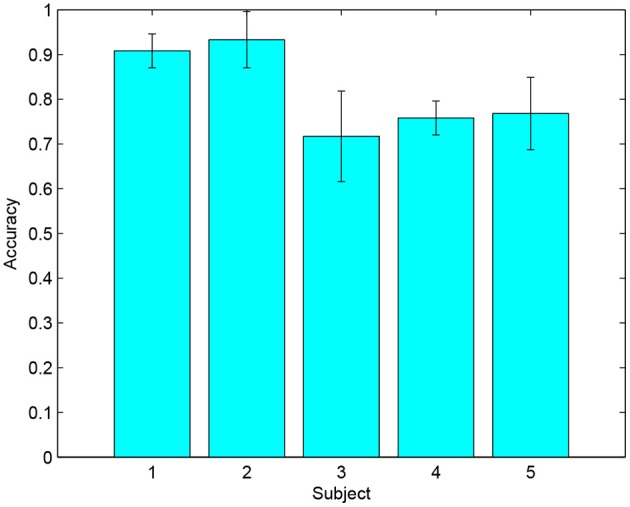
The average accuracies and standard deviations of the five selected subjects in the 9 MI training sessions.

According to the experimental results, five of the 22 subjects achieved a highest accuracy above 80% in an optimal session, as shown in Table 1. The average accuracies and standard deviations of these five subjects are presented in Figure 6. Among them, two subjects (S1 and S2) had prior experience with the MI paradigm, and the other three (S3, S4, and S5) were the first time to perform MI tasks. The highest MI accuracies for these five selected subjects in an optimal session were higher than 80%, and the average accuracies for each of them were higher than 70%. For the two subjects with prior MI experience, the highest accuracies were 95 and 100%, respectively. Except for the five selected subjects, six of the 22 subjects did not generate accuracy higher than random level (70% in this case, determined by the binominal test) in any session, which implied that no significant modulations of the sensorimotor rhythms were observed among them. The rest eleven subjects achieved a highest accuracy between 70 and 80% in an optimal session, which was higher than random level but might not be satisfied to realize a reliable control.

**Table 1:** Results of the five subjects in the MI-/EOG-Based sessions.

**Subjects**	**Gender**	**Age**	**MI accuracy (%)**	**EOG accuracy (%)**	**EOG RT (s)**	**EOG FPR (events/min)**
S1	Male	25	95	95	1.4	1.5
S2	Male	33	100	95.3	1.3	3.5
S3	Male	27	82.5	97.7	1.8	1.5
S4	Male	25	80	97.5	1.1	0.2
S5	Male	26	82.5	95.5	1.1	1
Mean ± SD	/	/	88 ± 8.9	96.2 ± 1.3	1.3 ± 0.3	1.5 ± 1.2

### 4.2. EOG Training Results

As shown in Table 1, all of the five subjects participated in this session could achieve an EOG accuracy (the highest value of all sessions) above 95% for the button selection task. The average EOG accuracy for these subjects was 96.2 ± 1.3%, which demonstrated that the individual variance of the proposed EOG paradigm was much smaller than that of the MI paradigm. According to the results, it took 1.3 ± 0.3 s in average to generate a command through the EOG paradigm, which was faster than that proposed in some EOG-based state-of-the-art works (typically 2–3 s) (Ma et al., 2015; Huang et al., 2018). In this study, the FPR was evaluated without the verification process, aiming to verify the effectiveness of the proposed method based on the peak amplitude and timing (see details in the *EOG signals processing* section to distinguish intended and unintended blinks. For these five subjects, the average FPR was 1.5 ± 1.2 events/min. Since a healthy person with normal eye movements usually performed 10–20 unintended blinks per minute, the probability that an unintended blink was mistakenly regarded as an intended one in this work was ~7.5–15%. This probability was considered to be acceptable since there was a verification process (i.e., raising eyebrows) after the blink recognition, which could ensure that the error recognition of unintended blinks would not result in any output command.

### 4.3. Mobile Self-Drinking Experiment Results

The average number of collisions for each subject in the three concatenated tasks (Task 1: reaching the table; Task 2: grasping the bottle to drink and put it back; Task 3: passing the door) in this experiment were illustrated in Table 2. For Task 2, a failed grasp was counted as a collision. According to the results, S1 successfully completed the three runs without any collision. S2 completed the first two tasks in all three runs but failed to pass through the door in one run. Thus, the average number of collisions for S2 to complete a run of Task 3 was around 0.3. S4 completed Task 1 without any collision, and generated 1/0.3 collisions averagely in Task 2/Task 3. For S3 and S5, the average numbers of collisions in Task 1 were 1.3 and 1.7, respectively, which might be resulted from the relatively unstable direction control of the wheelchair.

**Table 2:** Results of the mobile self-drinking experiment.

**Subjects**	**Task 1**	**Task 2**	**Task 3**
S1	0	0	0
S2	0	0	0.3
S3	1.3	0.3	0.7
S4	0	1	0.3
S5	1.7	1	0
Mean	0.6	0.5	0.7

## 5. Discussion

Previous wheelchair systems controlled by BCIs were generally tested by asking the subject to drive the wheelchair from one place to another without accurate requirements of the distance and direction control accuracy. In this study, the subjects need to accurately control and stop the wheelchair in front of a table with a certain distance range and direction. Otherwise the grasping task will fail. Thus, a main purpose of this work is to prove that a hybrid BCI can provide satisfied control precisions (both the distance and direction) for the wheelchair and the robotic arm to handle tricky daily tasks.

In this study, the moving task was concatenated with the grasping task. To ensure a successful grasp, the target bottle has to be located within a limited rectangular space 0.4 m ahead of Camera A (length: 0.8 m; width: 0.4 m; height: 0.6 m). Thus, the required positional accuracy of this task is 0.4 m. There are no reports of any BCI-controlled wheelchair systems achieving such accuracy. In this study, considering that the wheelchair speed is 0.2 m/s and the stop RT is ~1.15 s, the proposed system achieves a positional accuracy of 0.23 m, which is satisfied compared with the required accuracy. Moreover, since the proposed system generates nearly continuous directional control outputs, the user can accurately adjust the wheelchair to ensure it is facing almost directly to the target. According to the experimental results, three of the five subjects successfully completed the Task 1 of the mobile self-drinking experiment, and all of the five subjects completed Task 2 and Task 3 with no more than 1 collision in average, which demonstrated the proposed hBCI provided sufficient accuracies to control the integrated system.

In the proposed hybrid BCI, we attempt to use the EOG signals to handle the out-of-control problem of MI-based systems, which is caused by the time-varying characteristic of the EEG signals and is more serious in an integrated system task, since it usually consumed more time and efforts than a single device task does. Specifically, users could select the corresponding buttons through the EOG paradigm to renew the MI parameter *C*_*idle*_ using real-time EEG signals and adjust the left/right turning threshold *TH*_*l*_ and *TH*_*r*_. To renew *C*_*idle*_, the user first stopped the wheelchair and then perform eye movements to select the “Renew” button. After the button was selected, the user kept in idle state for at least 10 s. The algorithm averaged the scores during this period and used it as an offset compensation for *C*_*idle*_. Moreover, if some unreliable issues caused an offset in the left/right classification, the user could increase/decrease the left/right threshold with a step of 0.2 by selecting the “+”/“−” buttons on the wheelchair panel. According to the observations during the experiments, the three subjects without prior MI experience (S3, S4, and S5) could extend the time of effective control through this strategy, which supported that this strategy might be a feasible solution to utilize the reliability of EOG signals to overcome the time-varying characteristic of the EEG signals.

For subjects maintaining normal eye movements, EOG may be a better choice for developing HMIs since it usually has a higher signal-to-noise ratio. However, EOG-based HMIs can only provide discrete commands, which hurts the control precision in scenarios that require continuous control, such as the direction control of the wheelchair. Compared with EOG, the motor imagery (MI) paradigm used in BCI has a better real-time response performance (usually a few hundreds of millisecond). In this work, the timing window length of an EEG signal epoch for the MI classification was 2 s, and the interval between the starting points of two temporal adjacent epochs was 0.2 s (i.e., the algorithm generated a MI classification result for every 0.2 s). Moreover, the left/tigh threshold conditions were applied to further smooth the outputs. Other wheelchair commands and all of the robotic arm commands were generated by EOG.

Other functions of the wheelchair, such as moving forward/backward and stopping, can be realized in a discrete control mode. Thus, we used an EOG-based button selection paradigm similar with the one proposed in Huang et al. (2018). In Huang et al. (2018), users performed 3-4 blinks to select a button and resulted in a RT of 3.7 s. In this study, users performed one blink and one eyebrow movement for button selection, and the average RT was reduced to ~1.4 s. For the robotic arm, since the required positional accuracy of a grasping task usually reaches centimeter-level, it is challenging to use a single BCI to realize the full control of the arm. Therefore, we implemented a shared control mode to combine the intelligence of the robotic arm with the EOG paradigm. Once the user selects a button which represents a target bottle, the robotic arm automatically plans the path between the target object and the user's mouth according to the accurate coordinates obtained by the two cameras.

## 6. Conclusion

In this paper, a novel hBCI based on EEG and EOG was presented for the control of an integrated assistive system, which consisted of a wheelchair and a robotic arm, aiming to help users move from a random place and grasp a target object that is placed far away. Users steered the wheelchair left/right by performing motor imagery of the left/right hand, and generated other wheelchair or robotic arm commands by implementing two kinds of eye movements (blinking and raising eyebrows). Five subjects were asked to use the system to complete a mobile self-drinking experiment, which included several tricky tasks, such as avoiding obstacles, grasping a target bottle and passing through a door. The experimental results demonstrated that the proposed hBCI could provide satisfied control accuracy for controlling an integrated assistive system to complete complex daily tasks. In a future work, we will improve the hBCI paralyzed patients and expand its application range in the medical rehabilitation process.

## Data Availability Statement

The raw data supporting the conclusions of this manuscript will be made available by the authors, without undue reservation, to any qualified researcher.

## Ethics Statement

This study was carried out in accordance with the recommendations of the Ethics Committee Regulations, the Ethics Committee of Sichuan Provincial Rehabilitation Hospital with written informed consent from all subjects. All subjects gave written informed consent in accordance with the Declaration of Helsinki. The protocol was approved by the Ethics Committee of Sichuan Provincial Rehabilitation Hospital.

## Author Contributions

YL and QH came up with the main idea of this work, and QH realized it, completed the experiments, and wrote the paper with help from YL. ZZ and QH worked together to realize the pattern recognition part for the robotic arm control. TY provided kind help for building the MI training paradigm. SH provided the helpful advices for the hybrid wheelchair control strategy.

### Conflict of Interest

The authors declare that the research was conducted in the absence of any commercial or financial relationships that could be construed as a potential conflict of interest.
